# The connection between alexithymia and attachment style in the bipolar pathology

**DOI:** 10.1192/j.eurpsy.2025.1047

**Published:** 2025-08-26

**Authors:** C. Bredicean, D. Onita, Z. Popovici, F. Calota, A. Danciu, S. Ursoniu, C. Giurgi-Oncu

**Affiliations:** 1 Neuroscience, University of Medicine and Pharmacy Timisoara; 2 Psychiatry, Dr. Victor Popescu Emergency Military Clinical Hospital, Timisoara; 3 Psychiatry, The mental health center, Arad; 4 Functional Sciences, University of Medicine and Pharmacy Timisoara, Timisoara, Romania

## Abstract

**Introduction:**

Difficulties in identifying and expressing one’s emotions, known as alexithymia, may influence the development of bipolar disorder. Attachment style reflects how a person forms and maintains interpersonal relationships

**Objectives:**

The aim of the study is to analyze the connection between alexithymia, attachment style and the clinical evolution of bipolar disorder.

**Methods:**

The study was conducted on a sample of 31 subjects diagnosed with bipolar affective disorder, with a longitudinal evolution of at least 10 years. Sociodemographic parameters, alexithymia (Toronto Alexithymia Scale), and attachment style (Adult Attachment Scale) were evaluated. Data were collected and analyzed to identify potential correlations between levels of alexithymia and attachment styles, as well as their impact on the number of illness episodes.

**Results:**

There is no correlation between alexithymia and attachment style (r = -0.044, p > 0.05). A strong and significant correlation was observed between the level of alexithymia and the number of bipolar episodes (r= 0.907, p < 0.05). Attachment style did not have a significant influence on the course of illness. The educational level correlated inversely with alexithymia, but without being statistically significant (r=- 0.344, p > 0.05).

**Conclusions:**

Alexithymia has an important role in the evolution of bipolar disorder, as it appears to be associated with an increased frequency in the number of illness episodes. The development of psychotherapy interventions to reduce alexithymia are essential to ensure a satisfactory quality of life for patients.

**Disclosure of Interest:**

None Declared

